# “If you cannot measure it, you cannot improve it”. Outcome measures in Duchenne Muscular Dystrophy: current and future perspectives

**DOI:** 10.1007/s13760-024-02600-2

**Published:** 2024-07-31

**Authors:** Silvia Benemei, Francesca Gatto, Luca Boni, Marika Pane

**Affiliations:** 1 Medical Affairs, Pfizer Italy, Rome, Italy; 2 U.O. Epidemiologia Clinica, IRCCS Ospedale Policlinico San Martino, Genoa, Italy; 3 Nemo Clinical Centre, Fondazione Policlinico Universitario A. Gemelli IRCSS, Università Cattolica del Sacro Cuore, Rome, Italy

**Keywords:** Duchenne muscular dystrophy, Clinical outcomes, North star ambulatory assessment (NSAA), 6-Minute walking test (6MWT), 4 stairs climbed (4SC), Performance of the upper limb (PUL)

## Abstract

Duchenne Muscular Dystrophy (DMD) is an X-linked recessive neuromuscular disorder primarily affecting males, caused by mutations in the dystrophin gene. The absence of dystrophin protein leads to progressive skeletal muscle degeneration. Recent advances in the therapeutic landscape underscore the need to identify appropriate outcome measures to assess treatment efficacy in ambulant and non-ambulant DMD patients, across clinical and research settings. This is essential for accurately evaluating new treatments and attributing therapeutic benefits.

It is crucial to establish a robust correlation between outcome scores and disease progression patterns. This task is challenging since functional test performance may be influenced by different patient’s characteristics, including the physiological evolution of the neurodevelopment together with the disease progression. While widely used DMD outcomes such as the North Star Ambulatory Assessment, the 6-Minute Walking Test, the 4 stairs climbed, and the Performance of the Upper Limb exhibit reliability and validity, their clinical significance is influenced by the wide phenotype and progression variability of the disease.

We present and discuss the features (relevance, quantifiability, validity, objectivity, reliability, sensitivity, specificity, precision) of available DMD outcome measures, including new potential measures that may be provided by digital tools and artificial intelligence.

## Introduction

Duchenne Muscular Dystrophy (DMD), an X-linked recessive neuromuscular disorder due to the absence or deficiency of dystrophin protein, has a global incidence of 1 in 5000 live male births [1]. The absence of dystrophin produces progressive skeletal muscle degeneration and weakness, appearing before age 6 [2], leading to a loss of ambulation (LOA) and impairment of respiratory and cardiac muscles that is the main death cause [3].

Improvements of standard of care therapy, have significantly delayed LOA onset, from a mean age of 8.5 years before the advent of steroids, to 13 to 14 years in the last two decades [4]. Besides the administration of glucocorticoids the main treatment strategy for DMD currently involves a multidisciplinary approach [1].

Open therapeutic issues include cardiorespiratory complications, psychological and social disturbances, and the pediatric to adult care transition [5]. Indeed, DMD patients are at a high risk of developing anxiety and depression, cognitive function disorders, including intellectual disability, attention deficit hyperactivity disorder, obsessive-compulsive disorder [6, 7]. In addition, the transition to adult care is accompanied by challenges due to the complex healthcare needs associated with the progression of the disease [8, 9]. Notwithstanding the clinical variability, although the typical age of death was in late twenties, nowadays DMD patients have been surviving up to the 30s and beyond, with a median life expectancy of 28.1 years for patients born after 1990 [10]. Big therapeutic advancements, including gene therapy, are emerging, but the determination of the most suitable treatment for each patient remains challenging, given the disease high phenotypic variability and progressivity [11]. Understanding disease progression in research and clinical practice is also challenging because DMD can affect the normal growth and development of the brain, thus impacting physiological neurodevelopmental growth patterns. As a result, it is complicated to distinguish between impairments caused by the disease itself and those related to impaired developmental processes [1]. Indeed, mutated isoforms of dystrophin expressed in the brain cause modifications in the volume and organization of brain Purkinje cells, impacting synaptic neurotransmission with the possible development of cognitive deficits, impairment of learning abilities, and potential behavioral issues [1].

With the increased number of clinical trials in DMD over the past two decades [1], the dialogue between stakeholders (physicians, regulators, biostatisticians) has become crucial.

Much consideration has been given to outcome measures for treatment efficacy and safety, in clinical and research settings, in ambulant and non-ambulant DMD patients [12], to attribute appropriate value to the new therapies.

We aim to provide a comprehensive DMD outcome measure description and challenges related to their identification, optimization, validation and application, within the framework of clinical research methodology, taking into account the evolving therapeutic landscape and the more recent knowledge and technology advancements.

## Current classification of outcomes

Effective outcome measures stand as a cornerstone in evaluating therapies. Clinical trials may significantly impact patient care, prioritizing outcomes aligned with the everyday patient experience. Drawing a parallel with Galileo Galilei’s groundbreaking scientific revolution, it becomes evident that achieving remarkable results requires looking in the right direction (the sky) with the appropriate tools (telescope). In clinical trials, the right direction encompasses clinically meaningful outcomes, while the right tools consist of valid and significant endpoints. Unfortunately, numerous trials fall short in this regard, due to inadequacies in outcome selection, data collection, and reporting practices [13].

To be effective, outcome measures must possess specific attributes, as outlined by Piantadosi in 2005 [14] (Table 1), and it is imperative to understand the potential pitfalls that can undermine their validity.

**Table 1:** Endpoint characteristics according to modified Piantadosi’s criteria [14]

Characteristic	Meaning
Relevant	Clinically important/useful. The importance may be function of the development phase of the research. For example, for early developmental trials evidence of biological marker activity is usually sufficient for clinical relevance.
Quantifiable	Measured or scored on an appropriate scale, i.e. is able to be expressed as an amount, quantity, or numerical value
Valid	The measure adequately represents the underlying construct that it is supposed to measure
Objective	Interpreted the same by all observers, even if some clinical endpoints, such as symptoms, quality of life, and other patient-reported outcomes may and should contain a subjective component
Reliable	Same effect yields consistent measurements, i.e. under the same conditions the method can systematically reproduce the same results multiple times
Sensitive	Responds to small changes in the effect of the intervention
Specific	Unaffected by uncontrolled factors that can influence the results of a measure
Precise	Has small variability, i.e. the observed scores tend to be aggregated around their average value

Outcome measures can be categorized as biomarkers that measure biological phenomena (e.g., blood metabolites, electrophysiological markers, or imaging results), or clinical measures that assess meaningful aspects. In therapeutic trials, selecting outcome measures aligned with the clinical development phase is essential. Biomarkers prove most valuable in exploratory trials (Phase I, IIa), while clinical measures are better suited for efficacy trials (Phase IIb or III) designed to evaluate phenomena of primary interest for patients, such as survival, disease progression, and quality of life (QoL). In some scenarios, specialized assessments addressing disease-specific issues may be favored (e.g., targeting respiratory function is essential when respiratory complications arise). In the case of infants, toddlers, and young children, age-validated tools are deemed essential. Remarkably, regulatory authorities like the EMA and FDA although providing details about outcome measures and their applicability (Table 2), have not provided specific recommendation for the choice of measurement tools for specific DMD trials, as the choice must be tailored to study population and design.

**Table 2:** Current position of Regulatory Authority on outcome measures capturing treatment efficacy in DMD

Regulatory Authority document	Outcome measure in trials
FDA	No specific guidance
EMA Guideline on the clinical investigation of medicinal products for the treatment of Duchenne and Becker muscular dystrophy (Recommended by EMA CHMP) [40]	• Set of candidate outcome measures including NSAA, 6MWT, PUL (to be chosen on a case-by-case basis) according to (i) scientific question, (ii) patient age, (iii) disease stage, (iv) mechanism of action of the tested compound, (v) study duration • “*two endpoints should be selected from the domains muscle strength […] and motor function. According to the motor system parameter estimated to be particularly affected*,* one should be selected as primary endpoint and the other as secondary endpoint. Effects on the single selected primary endpoint should be supported by results from the most relevant secondary endpoints for consistency”*

### Challenges in selecting DMD Outcome measures

Several clinical challenges undermine the choice of outcome measures in DMD, including the clinical variability and stratification of phenotypes together with a lack of comprehensive knowledge on the disease natural history.

#### Variability in disease progression

In ambulant DMD patients, disease progression shows significant variability, ranging from potential improvements to rapid deterioration [15]. Genetic diversity among DMD patients is considerable and several cohort studies have sought to establish genotype-phenotype correlations with varying outcomes [15, 16]. Genotype differences influence long-term progression [17]; moreover, corticosteroids’ use further contributes to clinical heterogeneity, variably delaying the decline in muscular, pulmonary, and cardiac function [18, 19] with possible genotype-drug therapeutic interactions [20]. Additionally, several factors influence changes in functional tests over time, including age, baseline ambulatory function, variations in muscle load, stress, fiber-type composition and maturation [16, 17, 19, 21, 22]. Importantly, age per se offers limited prognostic value given the variability in disease progression and function with time [23].

The onset and progression of DMD outcomes do not consistently follow a stereotypical pattern. Cardiomyopathy, for instance, may occur earlier or later in some patients, and it does not always correlate with muscular weakness [24].

Efforts have been made to categorize DMD trajectories [23], and researchers are exploring magnetic resonance imaging (MRI) for muscle composition and other biomarkers to categorize the disease progression and to predict clinical outcomes [25].

Additionally, appropriate outcome measures to assess upper limb function and functional abilities across the ambulant to non-ambulant spectrum are required for older patients [26]. Signs of upper limb weakness can manifest early in ambulant DMD patients, while non-ambulant patients exhibit variability due to the LOA timing and the onset of scoliosis, osteoporosis, obesity, disuse atrophy, contractures, and psychosocial challenges [18, 27, 28].

Importantly, it has emerged that combining multiple functional measures yields a more accurate prognosis then relying on a single measure alone [22].

#### Challenges in biomarkers development

DMD clinical trials have encountered substantial hurdles, including the lack of reliable biomarkers of disease progression [29]. Biomarkers may be classified as: (i) susceptibility/risk, (ii) diagnostic, (iii) prognostic, (iv) disease progression monitoring, (v) predictive, (vi) pharmacodynamic, (vii) safety [30]. For the development of drugs, biomarkers are particularly relevant when they are validated as surrogate endpoints, and accordingly they may substitute a clinical endpoint [31]. Importantly, biomarkers that works as surrogate endpoints may contribute to speed up the definition of a drug benefit/risk balance thanks to a decreased variability in comparison to functional tests [29]. Similar to other rare diseases, the identification and validation of biomarkers for DMD is challenged from different points of view, including technical criticalities associated with the DMD multi-faceted pathophysiology, the limited number of samples mirroring the low number of patients, the limited applicability of statistical models developed for non-rare diseases, and, in the case of more advanced technology, the increasing costs and the complex management of high-throughput data [29]. Notwithstanding the abovementioned challenges, much attention continues to biomarker development for DMD and some of them, including muscular injury biomarkers, microdystrophin and MRI measurement, are commonly included as endpoints in clinical trials. Creatine kinase (CK) and other muscle proteins, initially increased, decline over time as the disease progresses, primarily reflecting early muscle mass loss. Hence, their utility in monitoring progression and therapy response diminishes as the disease advances [29]. Furthermore, CK specificity is limited due to its possible modification in different muscle-related disorders, age, metabolic changes, trauma, and physical exercise significantly impact its levels [29].

Muscle injury biomarkers that are stable over time could be explorative for assessing the effectiveness of dystrophin replacement and sarcolemma-stabilizing therapies in younger DMD patients [32]. Microdystrophin expression is another surrogate endpoint proposed for monitoring drug activity in trials investigating medications that target (micro)dystrophin expression [33]. However, there is the urgent need to identify clinically meaningful biomarkers applicable for drugs acting by different mechanisms of action.

MRI-based measurement of fat fraction has emerged as a valuable and objective non-invasive tool to monitor progression. Disease progression can include muscle necrosis and dysfunctional regeneration with the substitution of muscle tissue with fat and fibrotic material. Importantly, muscle fat fraction correlates with functional outcomes, predicting mobility decline before any functional test [34].

To enhance biomarker accuracy, some studies recommend transitioning from single biomarkers to biomarker “panels/signatures,” a key step toward precision medicine [29].

## Current scenario

In the assessment of motor dysfunction, the distinction between ambulatory and non-ambulatory patients’ performances is crucial, considering also that most ambulatory patients will later become non-ambulatory. Currently, measurement tools suitable for multicentric trials and, hence, widely employed include the Northern Star Ambulatory Assessment (NSAA) [35], the 6-minute walk test (6MWT) [36], the 4 stairs climb test (4SC) [37], and the performance of upper limb (PUL) which is the sole tool for both ambulatory and non-ambulatory patients [38]. Some additional timed functional assessments (Ten-Meter Walk-Run Test, Four-Square Step Test, Timed up and go test) are available [39] for evaluating neuromuscular conditions. Importantly, according to EMA guidelines [40], clinical trials should choose one of the abovementioned endpoints, often NSAA, as primary, nonetheless including one or more of the others as secondary or tertiary endpoints. Table 3 classifies the main available outcome measures according to Piantadosi’s criteria.

**Table 3:** Classification of outcome measures in DMD according to Piantadosi’s criteria [14]

	Relevant	Quantifiable	Valid	Objective	Reliable	Sensitive	Specific	Precise
Clinical outcome								
NSAA [35, 41, 78]	√ for ambulatory patients	√	√	N.D.	√	N.D.	√	X
6MWT [43]	√ for ambulatory patients	√	√	√	√	√	X	X
4SC [45]	√ for ambulatory patients	√	√	√	√	√	X	X
PUL [38]	√	√	√	N.D.	√	√	N.D.	N.D.
PedsQL [61]	N.D.	X	√	X	√	N.D.	X	N.D.
PARS-III [62]	N.D.	N.D.	√	X	√	N.D.	N.D.	N.D.
Cardiac MRI [54]	√	√	X	√	√	√	√	√
Muscle MRI [57]	√	√	X	√	√	√	√	√

N.D., not determined

### North star ambulatory assessment (NSAA)

The NSAA is a validated and reliable DMD-specific assessment scale [35, 41] (Table 4), recognized as a reference outcome measure for assessing the course of the disease and, in the last years, frequently chosen as the primary endpoint in clinical studies [41].

**Table 4:** Main tests used to evaluate outcome of DMD treatments

Type of outcome	Description
The North Star Ambulatory Assessment (NSAA) [23, 35, 41]	• Evaluates lower limb motor performance in 17 items, such as rising from the floor, sitting to standing, jumping, running, walking, and stair climbing • Designed for ambulant individuals of ≥ 5 years of age and for younger DMD boys, categorizing items by expected achievements within specific age groups and validating them accordingly • Item score: o 2 in case of no modification of the activity o 1 in case of activity changes, reaching the goal with no assistance o 0 in case of not achieving the activity goal without assistance
The 6-minute walking test distance (6MWT) [36, 43]	• Assesses the maximum walking distance in meters on a flat track within a standardized 6-minute time frame • Evaluates functional exercise capacity, fatigue disease progression in ambulatory DMD patients • The patient is allowed to stop during the test (this will reduce the impact of the fatigue) on the test results • Score: it reduces with disease worsening
Performance of the Upper Limb (PUL) [38]	• Assesses upper limb function (shoulder, upper arm, lower arm/fingers) and overall upper limb abilities, including daily activities that are identified as relevant by patients and clinicians • Includes: o 22 items with an entry item to define the starting functional level o 21 items subdivided into shoulder level (4 items), middle level (9 items) and distal level (8 items) dimension • Item score: o a low score on the entry item excludes performance of high-level items o scoring options vary across the scale between 0–1 and 0–6 according to performance o Each dimension can be scored separately with a maximum score of 16 for the shoulder level, 34 for the middle level, and 24 for the distal level o A total score can be achieved by adding the three level scores up to a max global score of 74

The NSAA, a practical test that can be easily completed in 10 min, allows an extensive assessment of motor performance domains, finding wide use in clinical trials, both blinded and open-label, and observational studies [34, 41, 42]. NSAA is quick, clear, easy to implement and specific, but per definition applicable only in ambulant patients. Some disagreement or inconsistency among the observers’ ratings or measurements has been reported, indicating that the scoring can be, at least partially, subjective. However, an appropriate training period for the observers is suggested to reduce interobserver variability [41]; no difficulties in performing each item and in obtaining adequate videos with a hand-held camera are reported, even after a short training session. It is worth noting that the extensive application of the NSAA, with its several items for different motor domains, may allow the identification of meaningful item subsets and contribute to the improvement of the area.

### Six minute walk test (6MWT)

The 6MWT (Table 4), although not specific for DMD, has demonstrated validity, sensitivity and reliability, is accurate, simple, well-tolerated (Table 3), and may be used as a primary clinical endpoint in ambulatory DMD trials [43, 44]. However, the results can be affected by several factors, such as age, height, and weight; in addition, the test do not provide information on the specific muscle groups affected by DMD.

### Four stairs climb (4SC)

The timed 4 stairs climb (4SC), is considered a valid, reliable and feasible non DMD-specific measure [45] of motor function useful to assess dynamic balance, functional abilities and falling risk in children with DMD [37, 43] (Table 4). It is used both in clinical trials and in clinical routine as it is cost-effective, although it may be not specific for muscle strength assessment and influenced by conditions such as weight gain [44, 46].

### Performance of the upper limb (PUL)

The Performance of the Upper Limb module (PUL), often used as the primary endpoint for non-ambulant patients [47] (Table 4), was developed and validated to evaluate upper limb function across the spectrum of ambulant and non-ambulant DMD patients [38]. This module, in its original PUL 1.2 version, has shown reliability and a correlation with the 6MWT in ambulant patients [48, 49]. It is also sensitive to differences in steroid regimes in non-ambulant DMD boys [47].

A revised version known as PUL 2.0 has been developed, improving the scoring system and item linearity, reducing the ceiling effect, and detecting significant changes over a 2-year period [50]. In ambulant boys, decreasing functional ability measured by the 6MWT correlates with PUL 2.0 changes [51].

### MRI outcomes

The use of imaging techniques has been increasingly explored [52], as mentioned above for MRI applied to detect and track early muscle-related alterations [25].

Cardiac magnetic resonance imaging (CMRI) detects myocardial fibrosis. Although further validation is required, CMRI is a promising tool to evaluate cardiac involvement in DMD [53]. CMRI, due to increased sensitivity, outperforms echocardiography in the early detection of DMD-related cardiomyopathy [54], that being clinically silent in the initial stages of the disease, progressively worsens and can ultimately lead to death [54, 55]. Despite the recognition of the importance of cardiovascular involvement, regulatory agencies have not yet approved any cardiac endpoint [56].

As mentioned above, the distinctive shift from skeletal muscle tissue to fat, accompanied by a decline in overall performance can be assessed through MR fat fraction analysis [34, 57]. MR application is less valuable in younger boys, as they have low fat fractions in early disease stages [34], and efforts to enhance reliability in this condition are ongoing [58]. A proposed correlation between MR fat fraction data and clinical outcomes such as 6MWT can provide information about how muscle characteristics influence disease trajectories [59]. This analysis highlights the potential strength of imaging biomarkers for diagnosis and prognosis, and pave the road for further application of algorithms and artificial intelligence (AI) to the existing data [59]. While MRI findings still require validation, their potential to advance the comprehension and management of DMD is unquestionable.

### Patient reported outcomes measures (PROMs)

Patient-reported outcome measures (PROMs) are gaining significance in clinical studies, reflecting the growing emphasis on the patient perspective.

Many PROMs assessing QoL in DMD patients lack robust evidence supporting their validity [60]. Only three validated and reliable Health-Related Quality of Life (HRQoL) instruments are available for DMD: the Pediatric Quality of Life Inventory (PedsQL), Personal Adjustment and Role Skills Scale, 3rd edition (PARS-III) [61, 62] and Pediatric Outcomes Data Collection Instrument (PODCI) [63].

While some studies have shown correlations between HRQoL measures and functional tests, this relationship may not hold when considering changes over 12 months [64]. Also, for PedsQL and PARS-III, some issues have been raising. The PedsQL shows limitations as it contains only few items specifically addressing how children adjust to chronic illness. Moreover, several items in the PedsQL are not related to specific behaviors, meaning that this tool might fail in capturing the factors relevant to a child’s experience with chronic illness [62]. A Rasch analysis has indicated that the PedsQL does not effectively quantify HRQoL in patients with DMD [62, 65]. In contrast, the PARS-III while being specifically designed to gauge psychosocial adjustment in individuals with chronic physical illnesses like DMD, lacks a specific cutoff score [62].

### Digital tools and artificial intelligence (AI)

Traditional clinical trial endpoints have not kept pace with the recent progress in therapies. Many of these endpoints, such as the 6MWT, have remained unchanged for decades, despite advances in medical technology [66].

However, digital biomarkers, defined as quantifiable data measured by digital devices, are increasingly being used as they can provide objective and reproducible data from patients’ daily lives, reducing errors associated with subjective assessments [58, 67]. These biomarkers often utilize digital sensors to monitor both lower and upper limb movements [67], making them suitable for assessing also non-ambulant patients [66]. A wearable device to quantify patient’s ambulation ability by the measure of the stride velocity 95th Centile has been recently validated by the EMA through comparison with 6MWT and NSAA. Stride velocity 95th Centile is now considered acceptable as a secondary endpoint for ambulant DMD patients aged 5 and above in clinical trials [68].

Despite their potential, many digital biomarkers are limited in scope, primarily focusing on lower-body performance. Additionally, they often replicate human observer biases and do not harness the full power of AI in healthcare [69].

Ricotti et al. recently developed the KineDMD biomarker, which leverages AI to collect and analyze whole-body movement data of patients with DMD. It focuses on natural movement behavior, providing a comprehensive and robust measure of motor capability. This biomarker can predict disease progression and potentially track therapy response [69], however, full validation in large population is still lacking.

## Discussion

Evaluating disease progression in DMD is a significant challenge due to the number of variables in action, including the heterogeneity in the severity and symptoms affecting this population, and the involvement of children and adults, as this implies that developmental maturation, puberty, and old age need to be considered.

Additionally, patients with DMD often exhibit neuropsychiatric issues that include cognitive impairment, deficit in attention and/or hyperactivity disorder, autism spectrum disorder, anxiety, and obsessive-compulsive disorder. These neuropsychiatric manifestations add complexity to the assessment and management of disease, significantly impacting the QoL of the patients [1] but also the sensitivity and precision of functional tests. Comprehensive neuropsychological evaluations and tailored interventions are then required to support both physical and mental health needs of these patients, but also to develop appropriate tools to monitor treatment outcomes.

Assessing a meaningful change in the employed outcome measures, which genuinely reflects the aspects of disease progression important to the patients and their families, is urgent and crucial for comprehending the results of clinical trials and designing well-powered studies [70, 71]. A critical point for DMD trials is certainly the duration of observation, as for many studies only short-term results (e.g., 48 weeks) are available. Importantly, given the established DMD variability, longer time is likely needed to adequately measure benefit. Time is a primary determinant of DMD clinical features as, although with somehow unpredictable patterns, it matters for functional loss. Importantly, as mentioned above, a significant number of individuals with DMD have cognitive and behavior issues that contribute to existing limitations in functional outcomes and act as confounders for the functional assessment. Considering the complexity of DMD natural history and limitations in the DMD drug assessment, innovative approaches are more than needed. The Critical Path Institute (c-path - DRSC consortia) has launched a clinical trial simulation platform, created from a series of disease progression models derived from the data in the database [72]. This international platform will allow clinical trial sponsors to forecast changes in clinically-meaningful endpoints, which would inform clinical trial protocol development with respect to inclusion criteria, endpoints, as well as the size and length and statistical analysis of clinical trials. Importantly, the Collaborative Trajectory Analysis Project has been extensively working around consensus models from aggregated data and pooled modeling in order to improve clinical trial design [42]. Further efforts are needed to improve the background knowledge needed to properly inform the design of clinical trials and improve their ability to detect the value of new treatments.

Meaningful differences in clinical presentation or disease progression and response to treatment involves two distinct approaches: Minimal Clinically Important Differences (MCID) and Minimal Detectable Change (MDC) methods. These two approaches are used in clinical research. Nonetheless, only a limited number of investigations have examined meaningful changes in various DMD outcomes [70, 71]. This scarcity might be attributed to the fact that many clinical trials primarily aim to decelerate disease progression rather than enhance motor function. Table 5 summarizes the approaches used in clinical research to determine meaningful differences in clinical presentation and response to treatment.

**Table 5:** Clinically meaningful changes. Approaches used in clinical research to determine meaningful differences in clinical presentation and response to treatment, ensuring that changes in clinical measures are statistically significant and clinically meaningful

Minimal Clinically Important Differences (MCID)	Minimal Detectable Change (MDC)
It is a measure of the minimum change in a patient’s score that is considered clinically meaningful. *“… smallest score difference in the relevant domain that patients consider beneficial*,* warranting a change in patient management*,* provided there are no troubling side effects or excessive costs***”** [79].	It quantifies the change needed to overcome measurement variability. It represents the minimum amount of change in a patient’s score that can be confidently attributed to a variation in their condition rather than a measurement error. Importantly, small changes may hold statistical significance just due to an exaggerated sample size, they may lack clinical relevance [80].

**Fig. 1:**
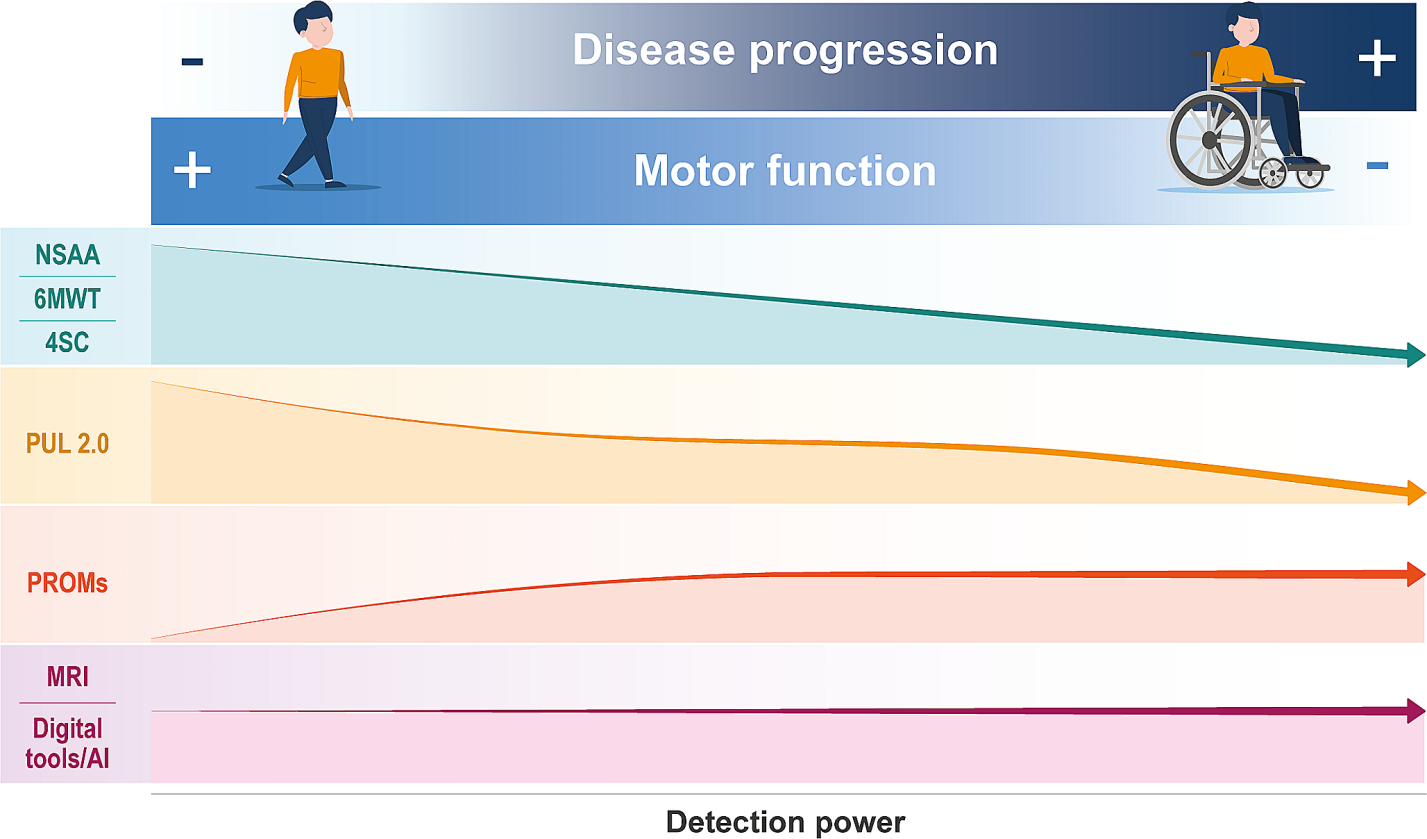
Qualitative scheme of the change of the detection power of the different outcome measures along with the DMD disease progression

Given the considerable variability in disease progression and phenotype, and the differences in patients’ ability to perform functional tests, it is imperative to establish a strong correlation between changes in outcome scores and the pattern of disease progression. Indeed, as the disease progresses and motor function declines, some of the traditional outcome measures become less effective at detecting functional changes. On the other hand, PROMs which may not detect differences in the early stages of the disease, become more valuable in assessing changes as the disease advances. Digital tools, also supported by AI, and bioimaging, based on objective measures, should provide an evaluation that is not influences by the progression of the disease (Fig. 1).

Some of the most used outcomes need improvements, as they were validated when the disease scenario was different. For NSAA, a linearized scoring system has been introduced to convert raw scores into a linear scale (0 to 100) [73], enhancing the psychometric robustness of scoring across a broader range of disease stages and is particularly useful for studies over 18–24 months [58].

Integrating patient’s perspectives with functional scores may increase the clinical meaningfulness of outcomes changes, differentiating between a reduced ability and complete loss of function [70]. Notably, this optimization of NSAA analysis reinforces the intrinsic potential of a multi-item score as outcome measure for complex disease like DMD.

The influence of age is significant for several outcomes: children < 7 years old may show increases in 6MWT over a year despite muscular impairment [74]. For older DMD subjects, rates of decline in 6MWT usually vary. Normative data across age groups, genders, and body sizes facilitate comparisons, acknowledging the fact that performance in healthy volunteers changes with development and aging [23]. Patient stratification according to age, baseline 6MWT, and corticosteroid use is crucial in the trials that use the 6MWT, and adjusting 6MWT to a percent predicted 6MWT could help distinguish normal growth from disease-related progression and treatment effects [75].

Additionally, maintaining a longitudinal history of outcome scores in each patient improves the disease progression evaluation. Muntoni et al. recently suggested that understanding individual NSAA skills and their scores at previous assessments can provide a more granular approach to assessing functional status [71].

Finally, when recording NSAA data, being valuable for any multi-item outcome [71], it is necessary to report explanations to clarify the score in case all the activities receive the “not obtainable” qualification, and to enhance the value of longitudinal data.

Linear outcomes for assessing upper limb function are strongly recommended, especially with the growing need to include older and non-ambulatory patients in clinical trials; importantly, factors like muscle contractures, Body Mass Index, and steroid variations may alter upper limb outcomes [58]. The relationship between PUL and overall functional abilities in ambulant patients is not linear, indicating that the rate of change is not constant across the three domains [51]. This non-linearity can be challenging for trial design, particularly when involving patients with varying functional abilities. Efforts are underway to predict the rate of progression for the individual domains and for the total score.

Many physical assessments heavily depend on a patient’s ability to cooperate with clinicians, follow instructions precisely, and execute the test. These assessments can be influenced by the patient’s motivation, level of physical activity, age, psychological well-being, and attention span, that may significantly impact the sensitivity and precision of functional tests. Ensuring consistency is of paramount importance, necessitating high standardization for the different evaluation tools, supported by top-quality training modules [58].

Despite its potential, imaging is not a routine part of the long-term clinical follow-up due to the lack of standardized protocols for image acquisition and data analysis, the high cost, and scan durations, which may hinder compliance in younger patients [58]. However, its role may increase in the future thanks to tech advancements. In this boosted tech scenario, digital sensors integrated with AI are emerging as promising tools for objectively and reproducibly capturing functional changes and for interpreting data from omics analyses. Advances in omics technologies generate unprecedented data for disease modeling. As proven by the AI-driven platform PandaOmics, which integrates multiple datasets to predict biomarkers and therapeutic targets based on disease relevance and clinical trajectory [76], AI and machine learning, aggregating disparate data types may identify complex interactions and characterize disease patterns with therapeutic potential beyond human capabilities [77].

In this scenario, PROMs are certainly valuable but they should not be expected to strictly correlate with functional changes, due to the possible adaptation of the individuals to their changed functional status and the high subjectivity of evaluation [58].

## Conclusions

Developing and optimizing outcome measures for specific clinical needs, requires extensive landscaping work and focusing on relevance to patients.

A better understanding of the relationship between clinical progression in DMD and endpoints within clinical trial timeframe would be helpful for drug development, is required by regulators, and needed by all stakeholders.

To this aim, establishing robust links between outcome scores and disease progression patterns is essential and may be favored by expressing scores as a percentage of healthy peers’ achievements, maintaining longitudinal score histories, and avoiding temporary null scores within multi-item outcomes.

The quote in the title is commonly cited as a summary of a more elaborate sentence by Lord Kelvin^1^, which ultimately attributes the meaning of scientific knowledge to both the measurability of phenomena and the ability to express, in numerical terms, what has been measured. This reflection suits perfectly with clinical research also in the DMD. Our ability to properly measure disease manifestations and to detect clinically meaningful changes related to treatments, will be the proof of a satisfactory knowledge that may open the road to further improvements in patients’ lives.

## Data Availability

No datasets were generated or analysed during the current study.

## Footnotes

1 *“I often say that when you can measure what you are speaking about*,* and express it in numbers*,* you know something about it; but when you cannot measure it*,* when you cannot express it in numbers*,* your knowledge is of a meagre and unsatisfactory kind; it may be the beginning of knowledge*,* but you have scarcely*,* in your thoughts*,* advanced to the stage of science*,* whatever the matter may be”* Thomson W. Constitution of matter. Electrical Units of Measurement. In: Company Ma, editor. Popular lectures and addresses in three volumes. London (UK)1889. p. 73 − 4.
