# Correction to “If you cannot measure it, you cannot improve it”. Outcome measures in Duchenne muscular dystrophy: current and future perspectives

**DOI:** 10.1007/s13760-024-02619-5

**Published:** 2024-09-02

**Authors:** Silvia Benemei, Francesca Gatto, Luca Boni, Marika Pane

**Affiliations:** 1 Medical Affairs, Pfizer Italy, Rome, Italy; 2 U.O. Epidemiologia Clinica, IRCCS Ospedale Policlinico San Martino, Genoa, Italy; 3 Nemo Clinical Centre, Fondazione Policlinico Universitario A. Gemelli IRCSS, Università Cattolica del Sacro Cuore, Rome, Italy

**Acta Neurologica Belgica**

10.1007/s13760-024-02600-2

The title of the article has been incorrectly stated as “Outcome Measures in Duchenne Muscular Dystrophy” in the original publication. The complete correct title should read as follows.

“If you cannot measure it, you cannot improve it”. Outcome measures in Duchenne Muscular Dystrophy: current and future perspectives

The original article has been corrected.

